# Transcriptome Analysis of Salt-Sensitive and Tolerant Genotypes Reveals Salt-Tolerance Metabolic Pathways in Sugar Beet

**DOI:** 10.3390/ijms20235910

**Published:** 2019-11-25

**Authors:** Gui Geng, Chunhua Lv, Piergiorgio Stevanato, Renren Li, Hui Liu, Lihua Yu, Yuguang Wang

**Affiliations:** 1 Key Laboratory of Sugar Beet Genetic Breeding of Heilongjiang Province, Crop Academy of Heilongjiang University, Heilongjiang University, Harbin 150080, China; genggui01@163.com (G.G.); yulihua@hlju.edu.cn (L.Y.); 2 Heilongjiang Sugar beet Center of Technology Innovation, Crop Academy of Heilongjiang University, Heilongjiang University, Harbin 150080, China; 3 Heilongjiang Provincial Key Laboratory of Ecological Restoration and Resource Utilization for Cold Region, College of Life Sciences, Heilongjiang University, Harbin 150080, China; lvchunhua1010@163.com (C.L.); lirenren529@163.com (R.L.); liuhui_lh128@163.com (H.L.); 4 DAFNAE, Dipartimento di Agronomia, Animali, Alimenti, Risorse Naturali e Ambiente, Università degli Studi di Padova, Viale dell’Università 16, Legnaro, 35020 Padova, Italy; stevanato@unipd.it

**Keywords:** salt stress, sugar beet, physiological analysis, transcriptomic analysis, differentially expressed gene

## Abstract

Soil salinization is a common environmental problem that seriously affects the yield and quality of crops. Sugar beet (*Beta vulgaris* L.), one of the main sugar crops in the world, shows a strong tolerance to salt stress. To decipher the molecular mechanism of sugar beet under salt stress, we conducted transcriptomic analyses of two contrasting sugar beet genotypes. To the best of our knowledge, this is the first comparison of salt-response transcriptomes in sugar beet with contrasting genotypes. Compared to the salt-sensitive cultivar (S710), the salt-tolerant one (T710MU) showed better growth and exhibited a higher chlorophyll content, higher antioxidant enzyme activity, and increased levels of osmotic adjustment molecules. Based on a high-throughput experimental system, 1714 differentially expressed genes were identified in the leaves of the salt-sensitive genotype, and 2912 in the salt-tolerant one. Many of the differentially expressed genes were involved in stress and defense responses, metabolic processes, signal transduction, transport processes, and cell wall synthesis. Moreover, expression patterns of several genes differed between the two cultivars in response to salt stress, and several key pathways involved in determining the salt tolerance of sugar beet, were identified. Our results revealed the mechanism of salt tolerance in sugar beet and provided potential metabolic pathways and gene markers for growing salt-tolerant cultivars.

## 1. Introduction

Soil salinization is one of the most common agricultural and environmental problems in arid and semi-arid regions of the world that seriously affects the productivity and quality of crops. Currently, approximately 950 million hectares of arable land are affected by salinity, 250 million hectares of which is irrigated land [1,2]. The utilization of saline soils and the breeding of salt-tolerant crops are, therefore, critical to guarantee an adequate food supply. Salt stress limits plant growth mainly through ionic toxicity and osmotic stress. The accumulation of ions (Na^+^ and Cl^−^) is toxic to cells by decreasing the water potential and stomatal conductance. At the same time, a low water potential in saline soil makes it difficult for the roots of plants to absorb water, causing physiological drought [3]. However, in order to survive under salt stress, plants adapt a series of complex strategies, including the efflux of ions, increased antioxidant enzyme activity and synthesis of osmotic adjustment proteins to cope with the stress. 

Halophytes, plants that survive to reproduce in environments where the salt concentration is around 200 mM NaCl or more, constitute about 1–2% of the world’s flora [4]. Halophytes are found in a wide range of angiosperm families and they occupy diverse habitats worldwide. Salt tolerance has evolved many times independently in different angiosperm lineages. Furthermore, evolutionary pattern of salt tolerance varies across angiosperm families, and salt tolerance can evolve frequently in the majority of families [5]. *Chenopodiaceae,* a typically Mediterranean species, is a xero-halophyte [6]. Sugar beet (*Beta vulgaris* L.), a dicotyledonous plant of the genus *Beta* in *Chenopodiaceae*, is the second largest sugar crop in the world. A wild ancestor of sugar beet is sea beet [*B. vulgaris* ssp. *maritima* (L.) Arcang.], which grows naturally along the Atlantic coasts of Western Europe and the coasts of Mediterranean countries [7]. To survive in these habitats, sea beet has developed structural and physiological strategies (i) to regulate distribution of salt and other solutes and (ii) to increase water content [8]. Many sugar beet cultivars inherited the salt tolerance trait from their ancestor and can complete its life cycle in soil containing 140 mM NaCl [9]. However, high levels of salt can significantly affect photosynthesis and decrease crop yield. Cultivating salt-tolerant sugar beet in saline soil is thus an effective way to increase production. In addition, identifying the mechanism of salt tolerance and the key genes implicated in salt tolerance will greatly accelerate the process of growing salt-tolerant cultivars.

Transcriptome analysis, a useful tool for the quantification of mRNA expression levels based on next-generation DNA sequencing technology, can identify key genes participating in various biological processes. Transcriptional analysis of common soybean under salt stress showed that 6422 and 4555 unigenes were differentially expressed in leaf and root tissues, respectively [10]. A comparative transcriptome analysis of *Brassica napus* roots was conducted between salt and control treatments showing that 163 genes were differentially expressed in response to salt stress [11]. Previously, we conducted the selection and cultivation of sugar beet salt-tolerant and salt-sensitive varieties. Our result demonstrated that T710MU is a salt-tolerant cultivar, while S710 is salt-sensitive. Two sugar beet genotypes have common origins, because T710MU and S710 have the same original male and female parents. After several years of screening under different salt conditions, salt-tolerant and salt-sensitive genotypes are selected, and the phenotypes can be inherited stably through self pollination, respectively. We hypothesized that transcriptome changes play an important role in sugar beet salt tolerance, and several genes or metabolic pathways may show different change patterns between two genotypes under salt stress. In this study, transcriptome sequencing using the Illumina sequencing platform was applied to identify changes in gene expression at the RNA level in sugar beet cultivars S710 (salt-sensitive) and T710MU (salt-tolerant) under salt stress, followed by a comparison of the salt-response transcriptomes in contrasting genotypes. Several physiological indicators, such as endogenous osmotic molecules and antioxidant enzyme activity, were also investigated. By identifying the transcripts that changed between the two genotypes under salt stress, we could acquire several key pathways involved in determining salt tolerance of sugar beet. Our results provide a comprehensive overview of the functional significance of several differential response genes in the establishment of an adaptive response to salt stress.

## 2. Results

### 2.1. Physiological Response to Salt Stress in Two suGar Beet Genotypes

**Figure 1:**
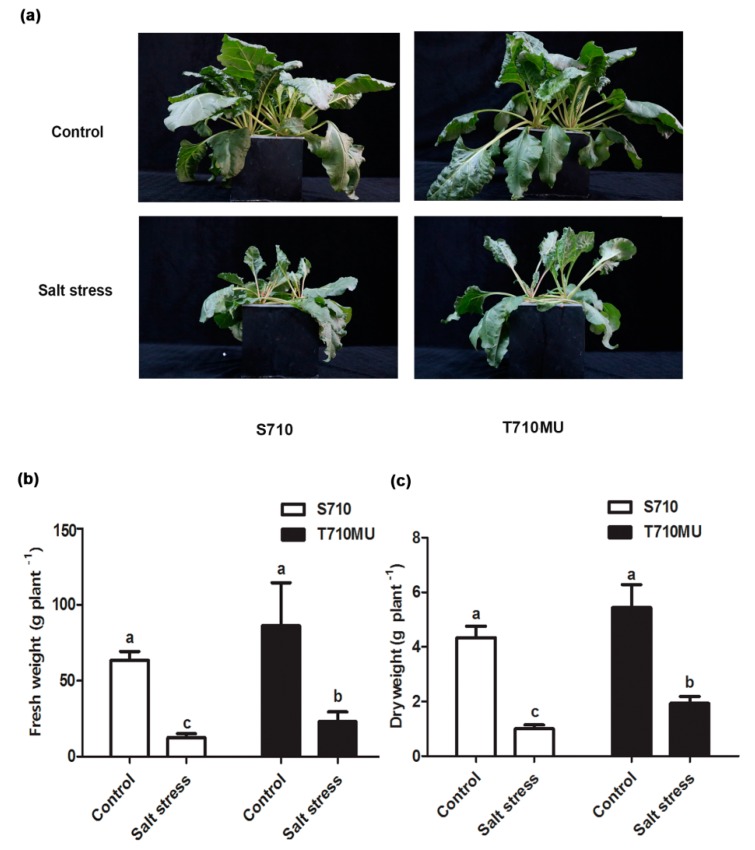
Morphological and physiological changes in sugar beet (S710 and T710MU) under control (0 mM NaCl) and salt stress (280 mM NaCl) conditions. Seven-day-old seedlings were treated with 280 mM NaCl for 15 days. Growth performance (**a**), fresh weight (**b**) and dry weight (**c**) were measured. Different letters indicate significantly different at *p* < 0.05. Three biological replicates were performed.

**Figure 2:**
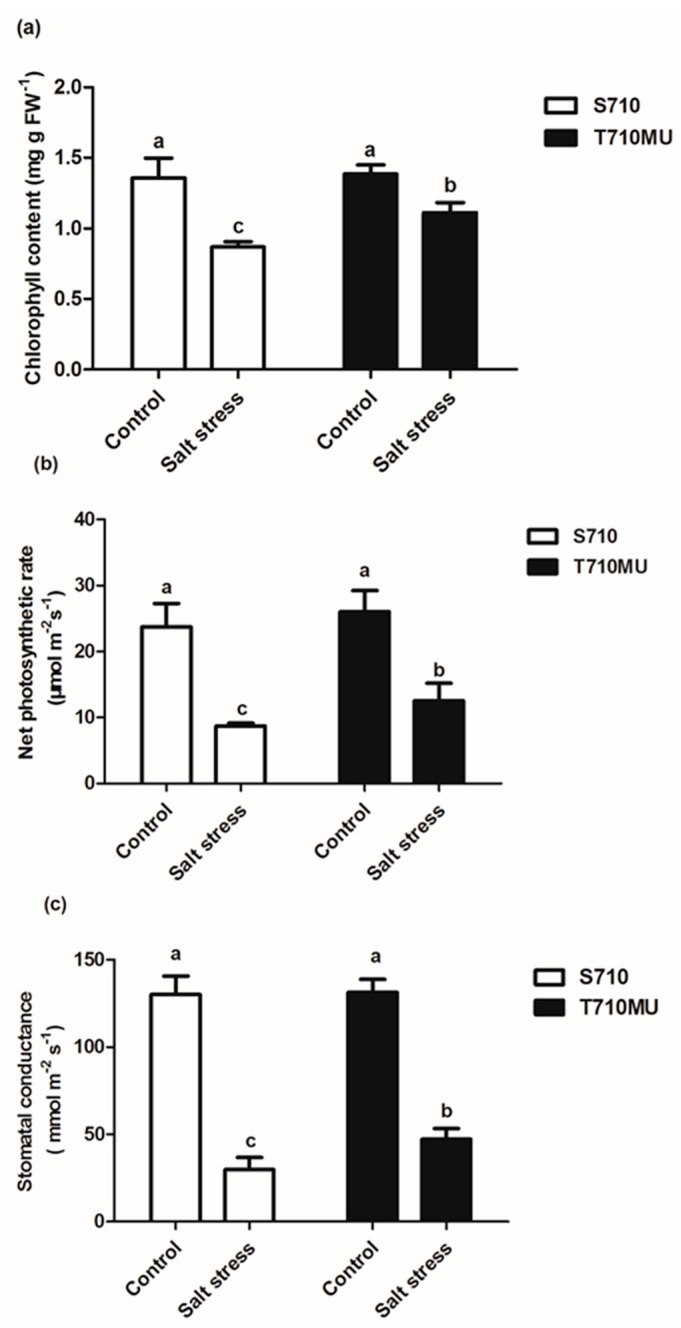
Effects of salt stress on photosynthesis-related parameters in the leaves of two sugar beet genotypes. chlorophyll content (**a**), net photosynthetic rate (**b**) and stomatal conductance (**c**) were measured. Different letters indicate significantly different at *p* < 0.05. Three biological replicates were performed.

We had previously identified salt-tolerant cultivars T710MU and salt-sensitive S710 at 280 mM NaCl. In this study, there was no difference between these two cultivars exposed to control conditions. However, salinity decreased the fresh and dry weights of both, which was particularly evident in the cultivar S710 compared to the T710MU one (Figure 1a). Its seedling fresh and dry weights decreased by 80.4% and 76.8%, respectively, after the plants were exposed to a high salt environment (Figure 1b,c). Instead, the seedling fresh and dry weights of cultivar T710MU only decreased by 73.4% and 64.5%, respectively, under salt stress (Figure 1b,c). The chlorophyll content and net photosynthetic rate are also photosynthesis-related indicators for evaluating the tolerance of plants to salt stress. In our study, salt stress significantly reduced chlorophyll content, net photosynthetic rate and stomatal conductance in both cultivars, but the values were much higher in cultivar T710MU than in S710 (Figure 2a–c). These results indicated that cultivar T710MU showed strong salt stress tolerance compared with the salt-sensitive cultivar S710.

**Figure 3:**
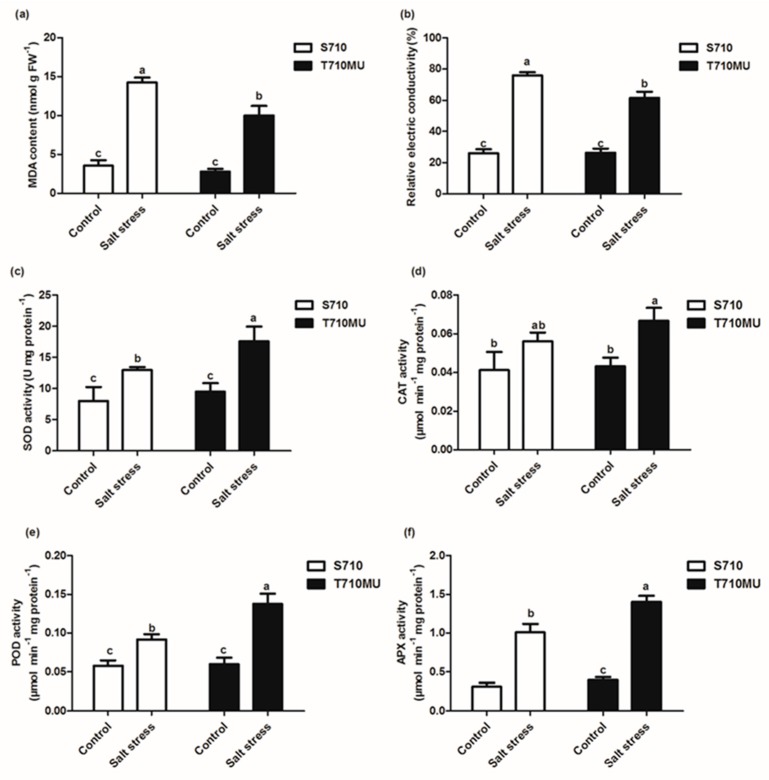
Effects of salt stress on antioxidant enzyme activity in the leaves of two sugar beet genotypes. The malondialdehyde (MDA) content (**a**), relative conductivity (**b**), superoxide dismutase (SOD) (**c**), catalase (CAT) (**d**), peroxidase (POD) (**e**) and ascorbic acid peroxidase (APX) (**f**) activity in sugar beet treated with 0 mM and 280 mM NaCl. Different letters indicate significantly different at *p* < 0.05. Three biological replicates were performed.

Accumulating reactive oxygen species (ROS), induced inevitably by salt stress, can damage the plasma membrane and increase membrane permeability. The malondialdehyde (MDA) content is a key indicator of the degree of lipid peroxidation. The MDA content in the leaves of seedlings exposed to salt stress increased significantly in both cultivars, but the MDA content was lower in the salt-tolerant cultivar T710MU than in the salt-sensitive S710 (Figure 3a). Furthermore, the relative conductivity was used to assess the degree of membrane permeability, as a higher conductivity is indicative of increased membrane permeability. Relative conductivity in the leaves of the salt-tolerant cultivar T710MU was lower than that in the salt-sensitive S710 (Figure 3b). Our results thus demonstrate that cultivar T710MU exhibited moderate lipid peroxidation and membrane damage under salt stress compared to cultivar S710. 

The antioxidant enzyme system in plants plays important roles in removing ROS and protecting cells from oxidative damage. The activities of superoxide dismutase (SOD), catalase (CAT), peroxidase (POD), and ascorbate peroxidase (APX) in both cultivars were determined under control and high salt conditions (Figure 3c–f). When plants were exposed to 280 mM NaCl for 15 days, antioxidant enzyme activity of both cultivars increased compared with plants exposed to control conditions. This phenomenon indicates that excessively produced ROS can activate the antioxidant enzyme system, which is a common response mechanism to salt stress in sugar beet. However, the activities of SOD, POD and APX in the leaves of cultivar T710MU were markedly higher than those in the leaves of S710 under salt stress (Figure 3c,e,f). These results indicate that cultivar T710MU showed strong antioxidant activity to cope with salt stress.

**Figure 4:**
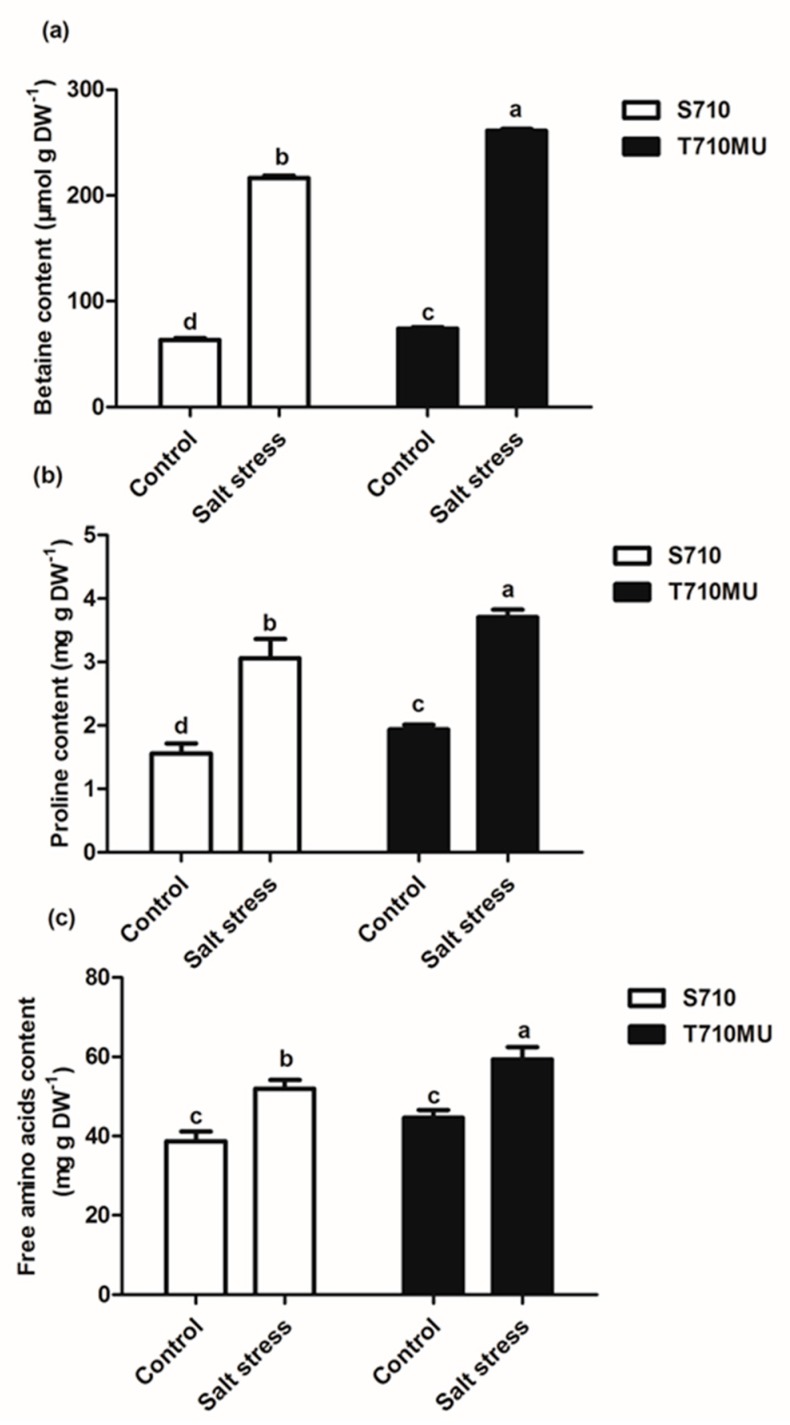
Effects of salt stress on the levels of osmotic adjustment substances in the leaves of two sugar beet genotypes. The levels of betaine (**a**), proline (**b**) and free amino acids (**c**) in sugar beet treated with 0 mM and 280 mM NaCl. Different letters indicate significantly different at *p* < 0.05. Three biological replicates were performed.

Plants can synthesize and accumulate osmotic adjustment molecules to maintain cell turgor and osmotic equilibrium in different compartments, especially when they are exposed to salt stress. In this study, the levels of three osmotic adjustment molecules increased significantly under salt stress (Figure 4). However, the levels of betaine, proline, and free amino acids in cultivar T710MU were notably higher than those in S710 under salt stress. The concentrations of betaine, proline, and free amino acids in the salt-tolerant cultivar were 20.52%, 31.21% and 24.81% higher than those in the salt-sensitive one, respectively. Salt-tolerant sugar beet, therefore, tends to synthesize higher levels of soluble organic solutes to improve its resistance to salt stress. 

### 2.2. Transcriptome Sequencing and Data Analysis

Leaf samples from the two cultivars exposed to control and high salt conditions (280 mM NaCl) were used to construct 12 libraries for sequencing (Table S1). Approximately 45,801,074 to 54,808,236 raw reads from each of the 12 libraries were acquired with 150 bp for both paired ends, and the Q30 of the base ratio was higher than 89.10%. After removing the low-quality reads, between 78.44% and 80.3% of the clean reads could be mapped to the sugar beet reference genome (version RefBeet-0.9; http://bvseq.molgen.mpg.de) (Table S1). These genes were used for further analyses.

As two sugar beet genotypes have common origins, we compared the SNPs that might be involved in different responses to salt stress in S710 and T710MU genotypes. This study compared S710 and T710MU genotypes with the reference sequence. We identified 3793 and 11,208 SNPs in cultivars S710 and T710MU, respectively (Tables S2 and S3). In addition, although T710MU has a close relationship with S710, many SNPs were only detected in the salt-tolerant cultivar T710MU. In total, 8947 SNPs were only characterized in the salt-tolerant cultivar T710MU (Table S4). These SNPs may participate in determining the sugar beet tolerance to salt stress. In the future, we will systematically study the relationship between these SNPs and sugar beet salt tolerance.

### 2.3. Identification of Differentially Expressed Genes

**Figure 5:**
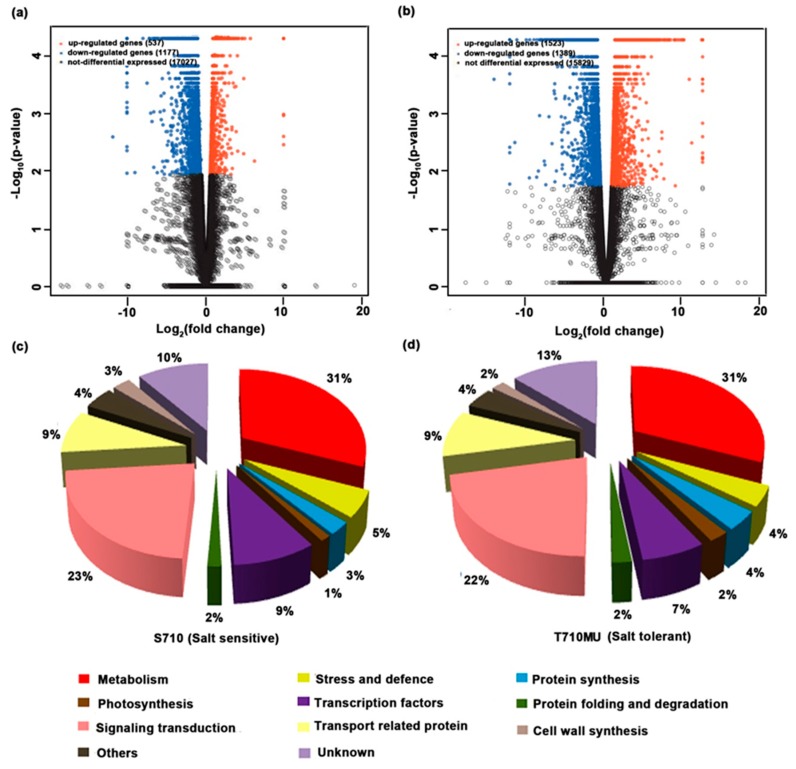
Volcano-plots and functional classification analysis of differentially expressed genes (DEG_S_) in the leaves of two sugar beet cultivars under salt stress. DEGs in the leaves of cultivars S710 (**a,c**) and T710MU (**b,d**). Each dot in panel a and b represents a specific gene or transcript, the red dots represent genes that were significantly up-regulated, the blue dots genes that were significantly down-regulated, and the black dots non-significantly changed DEGs.

To explore the molecular mechanism of the salt-tolerant cultivar and select potential markers for evaluating sugar beet tolerance, differentially expressed gene (DEG) analysis in the leaves of salt-sensitive and salt-tolerant cultivars under salt stress was conducted using the RNA-Seq method. To estimate the gene expression levels, the sequencing reads were mapped to the reference genome of sugar beet, and the fragments per kilobase of exon model per million fragments mapped (FPKM) value of each gene was calculated. All the annotated genes that were used to further identify DEGs in cultivars S710 and T710MU are listed in Tables S5 and S6, respectively. In our study, we used more stringent criteria (FDR < =0.05 and log2 fold change > = 1 and FPKM > =2) with three biological replicates to screen DEGs. In the salt-sensitive cultivar S710, 1714 DEGs were found to respond to salt stress (Table S7). The up-regulation of 537 genes and the down-regulation of 1177 genes were detected in the cultivar (Table S7 and Figure 5a). With regard to the salt-tolerant cultivar T710MU, 2912 genes were identified as salt stress responsive DEGs with 1523 up-regulated and 1389 down-regulated genes (Table S7 and Figure 5b). 

To determine the biological functions associated with DEGs in the salt stress response, the DEGs in leaves of the two cultivars, were classified into ten functional categories based on their basic functions (Figure 5c,d). The leaf salt stress-responsive genes in the salt-sensitive cultivar S710 were mainly categorized into metabolic processes (31%), signal transduction (23%), transcription factors (9%), and transport processes (9%). Similarly, in the salt-tolerant cultivar T710MU, the proportion of DEGs related to metabolic processes was the highest (31%), followed by signal transduction (22%), transport processes (9%) and transcription factors (7%). In addition, the proportion of DEGs related to protein synthesis in the salt-tolerant cultivar T710MU was much higher than that in the salt-sensitive S710. These results indicate that the functional classification of DEGs was similar between the two cultivars under salt stress.

### 2.4. KEGG Enrichment Analysis of DEGs

**Figure 6:**
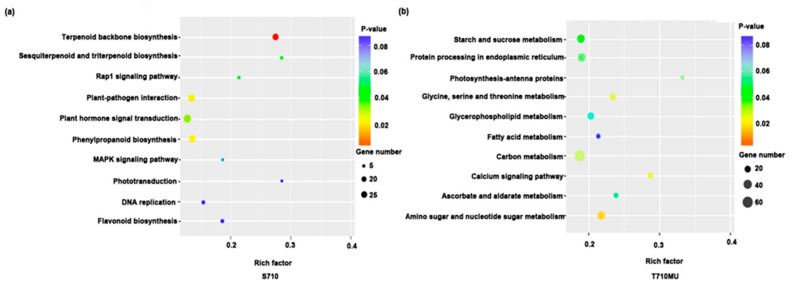
Top 10 KEGG pathways in the leaves of sugar beet cultivars S710 and T710MU under salt stress. KEGG enrichment pathways in S710 (**a**) and T710MU (**b**) under salt stress.

To identify the metabolic pathways involved in response to salt stress, we further analyzed the Kyoto Encyclopedia of Genes and Genomes (KEGG) enrichment pathways. Several metabolic pathways were significantly enriched in the two cultivars under salt stress. In S710, terpenoid backbone biosynthesis (*p* = 0.0009), plant-pathogen interaction (*p* = 0.027), phenylpropanoid biosynthesis (*p* = 0.0321) and plant hormone signal transduction (*p* = 0.0482) were the significantly enriched pathways (Figure 6a). Instead, in T710MU, the most significantly enriched pathway was amino sugar and nucleotide sugar metabolism (*p* = 0.0218), followed by calcium signaling pathway (*p* = 0.0261), glycine, serine, and threonine metabolism (*p* = 0.0285), carbon metabolism (*p* = 0.0313), protein processing in endoplasmic reticulum (*p* = 0.0304), and starch and sucrose metabolism (*p* = 0.0414) (Figure 6b).

### 2.5. Amino Sugar and Nucleotide Sugar Metabolism, C-Alcium Signaling Pathway and Glycine, Serine and Threonine Metabolism Are Involved in Sugar Beet Salt Tolerance

Compared with samples from salt-sensitive S710, we found many genes involved in amino sugar and nucleotide sugar metabolism were up-regulated in cultivar T710MU but there was no change in cultivar S710. For example, *UDP-D-xylose synthase*, *galacturonosyltransferase*, *UDP-glucose 6-dehydrogenase*, *chitinase 6-like* and *UDP-xylose 4-epimerase* were all only up-regulated in T710MU (Table 1). Moreover, several DEGs related to calcium signaling pathway also showed different expression patterns in the two cultivars under salt stress. In calcium signaling pathway, three *calcineurin B-like*, two *phosphoinositide phospholipase C 4-like*, and one *calmodulin* were only up-regulated in T710MU, but there was no change in the salt-sensitive cultivar S710 (Table 1). In addition, some genes enriched in glycine, serine and threonine metabolism were also only dramatically increased in the salt-tolerant cultivar T710MU under salt stress. *Amine oxidase* participated in threonine metabolism (Table 1). *Serine racemase*, *tryptophan synthase beta chain 1-like* and *serine hydroxymethyltransferase 4* were involved in serine metabolism (Table 1). *Betaine-aldehyde dehydrogenase family 7*, *Choline monooxygenase*, and *glycine cleavage system H protein* take part in glycine metabolism (Table 1). Our results indicated that the tolerance of sugar beet was involved in these pathways.

### 2.6. Validation of Illumina Expression Patterns by qRT-PCR Analysis

**Figure 7:**
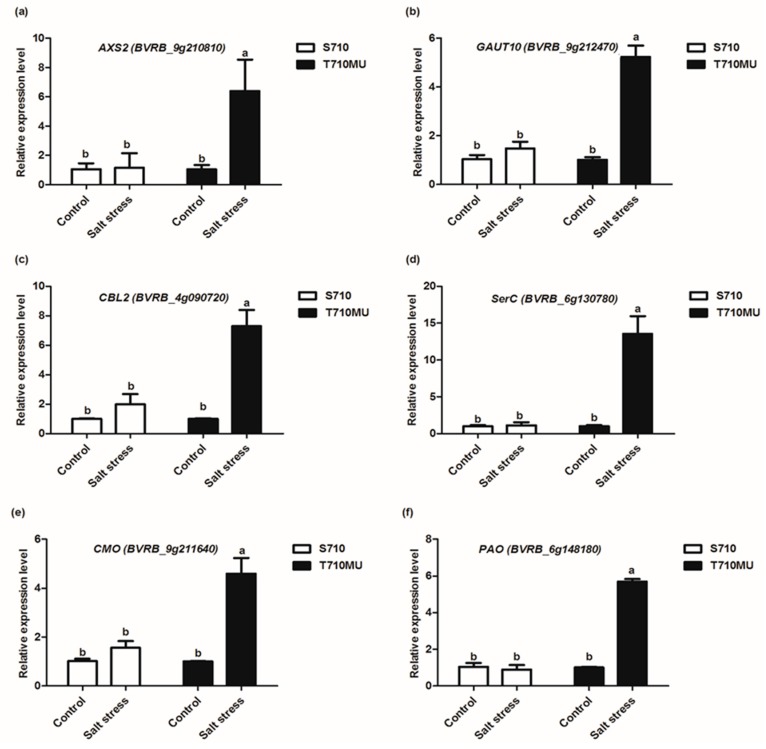
Relative expression of genes involved in salt stress in two sugar beet cultivars examined using RT-qPCR. The expression of *UDP-D-xylose synthase 2 (AXS2)* (**a**)*, Galacturonosyltransferase 10 (GAUT10)* (**b**)*, Calcineurin B-like protein 2 (CBL2)* (**c**)*, Phosphoserine aminotransferase (SerC)* (**d**)*, Choline monooxygenase (CMO)* (**e**) and *Primary amine oxidase* (PAO) (**f**) were detected. Different letters indicate significantly different at *p* < 0.05. Three biological replicates were performed.

To validate the DEGs acquired by the RNA-Seq method, the transcriptional levels of six genes were evaluated by qRT-PCR. Six genes were selected being representative of key point genes in amino sugar and nucleotide sugar metabolism, calcium signaling pathway and glycine, serine and threonine metabolism pathways involved in stress tolerance. The relative expression levels of the six genes from qRT-PCR are shown in Figure 7. Our results showed that in the salt-tolerant genotype, the mRNA expression of these genes were significantly changed in leaves upon salt stress, whereas in the salt-sensitive genotype, the expression of six genes were no changed in leaves in response to salt stress. The expression patterns of the most selected genes analyzed by qRT-PCR were similar to those obtained by the RNA-Seq method, although there were variations in the fold-changes (Table 1). These results, therefore, demonstrate that RNA-Seq analysis is a reliable tool to explore DEGs involved in the tolerance of sugar beet to salt stress. 

## 3. Discussion

Salt stress is an important limiting factor in the environment, and excessive amounts of Na^+^ and Cl^−^ adversely affect the ability of plants to take up water and nutrients, thereby causing imbalances in osmotic potential and ion levels. Sugar beet is a salt-tolerant crop. A transcriptome study has previously been performed in sugar beet M14. M14 is an interspecific progeny generated by crossing the cultivated sugar beet with the wild species *B. corolliflora* Zoss, which contains a wild chromosome [12]. In this study, we integrated physiological and transcriptomic methods to study the salt stress response mechanism of sugar beet cultivars S710 (salt-sensitive) and T710MU (salt-tolerant). The salt-tolerant cultivar T710MU showed better growth performance under salt stress, which included higher fresh and dry weights compared to the salt-sensitive S710. 

Chlorophyll, the main pigment of photosynthesis in plants, is located in thylakoid membranes. It plays important roles in light absorption and energy transfer. Several studies have shown that the chlorophyll content and net photosynthetic rate in plants decreased under salt stress [2]. It was also reported that chlorophyll content is positively correlated with the tolerance of plants to salt stress [13]. Stomata play a critical role in the regulation of gas exchange between the interior of the leaf and the exterior environment and are affected by environmental stimuli. Meanwhile, the valve of stomatal conductance was also correlated with the net photosynthesis rate. Net photosynthetic rate usually reflects the organic matter accumulated by plant photosynthesis, which is closely related to the dry matter accumulation per unit leaf area. In this study, we observed that the chlorophyll content and net photosynthetic rate of two sugar beet cultivars decreased, more so for S710 than for T710MU (Figure 2). These results indicate that cultivar T710MU exhibited a strong tolerance to salt stress by maintaining high levels of photosynthetic rate. 

Osmotic adjustment and solute accumulation play critical roles in the adaptation of plants to salt stress mainly by increasing the cytoplasmic concentration of osmotic adjustable organic substances, decreasing water potential and enhancing water uptake. Compared with the control, the levels of betaine, proline and free amino acids were increased in both cultivars under salt stress. However, these levels were higher in the salt-tolerant cultivar T710MU than those in the salt-sensitive S710 (Figure 3). Previously, it is found that under strong salinity proline content increased 1.7-fold in the halophytic beet but in sugar beet stayed at the control level [14]. These results show that the accumulation of osmotic regulatory substances plays a key role in determining the salt tolerance of sugar beet. 

Recently, a comparative transcriptome analysis between salt-tolerant and salt-sensitive jute (*Corchorus* spp.) genotypes in leaf and root tissues under salt stress and control conditions were conducted [15]. Methionine metabolism pathway was only enriched in salt-tolerant jute root tissue under salt stress, and it may be primary contributors to the resistant response in salt-tolerant jute [15]. Furthermore, transcriptome analysis of two contrasting sesame genotypes differing in salt tolerance was used to decipher the adaptive responses to salt stress. Several pathways were found to be preferably enriched with differentially expressed genes in salt-tolerant genotype, including alanine, aspartate and glutamate metabolism and carotenoid biosynthesis [16]. Metabolome profiling under salt stress showed a higher accumulation degree of metabolites involved in stress tolerance in salt-tolerant sesame genotype, and further highlighted that the amino acid metabolism, and sucrose and raffinose family oligosaccharides metabolism were enhanced in salt-tolerant genotype [16]. As sea beet displays elevated salt-tolerance compared to the cultivated beet, a study also investigated the transcriptomic responses to acute salt stress in sugar beet and its wild ancestor sea beet. Amino acid metabolism, secondary metabolism and photosynthesis were characteristic for sea beet, while carbohydrates metabolism, protein processing, pathogen response, transcription regulation and signaling as well as cell wall plasticity were specific for sugar beet [14]. In this study, to comprehensively analyze the mechanism of salt stress tolerance, transcriptome analysis of the two sugar beet cultivars with contrasting genotypes was performed using the high-throughput RNA-Seq approach. This analysis identified 4626 salt-responsive genes involved in stress and defense, metabolic processes, signal transduction, transport processes, and cell wall synthesis. Moreover, many of genes enriched in amino sugar and nucleotide sugar metabolism, calcium signaling pathway and amino acid metabolism showed different expression patterns in the two genotypes under salt stress (Table 1), and these pathways could participate in regulating the tolerance of sugar beet to salt stress.

### 3.1. DEGs Involved in Amino Sugar and Nucleotide Sugar Metabolism

Amino sugar and nucleotide sugar metabolism products have many roles in higher plants, in particular, they are important for cell wall synthesis and lesion repair. It was previously reported that amino sugar and nucleotide sugar metabolism was involved in the process of plant response to phytopathology, and plant-fungus interactions could hamper amino sugar and nucleotide sugar metabolism and impair pivotal functions in the plant host [17]. In this study, the expression of several genes related to amino sugar and nucleotide sugar metabolism was only induced in the salt-tolerant cultivar (Table 1). UDP-D-xylose synthase catalyzed the conversion of UDP-D-glucuronate to UDP-D-apiose. UDP-D-apiose was involved in the biosynthesis of D-Apiose that serves as an attachment point for borate cross-linking of rhamnogalacturonan II (RG-II) in the cell walls. Previously it was found that gene silencing of UDP-D-xylose synthase in plants caused a severe growth defect and cell death [18]. The increasing expression level of UDP-D-xylose synthase in T710MU was likely involved in maintaining normal wall structure under salt stress. Furthermore, five *galacturonosyltransferase* (*GAUT*) genes were up-regulated in cultivar T710MU under salt stress, whereas gene expression in the salt-sensitive S710 remained unchanged (Table 1). GAUT participate in regulating in cell-wall pectin and hemicellulose biosynthesis [19]. Recently, the increase in the numbers of *GAUT* from higher plants indicates that these genes play a key role in plant adaptation to living on land [20]. It was reported that *OsGAUT21* was significantly up-regulated under cold stress and participated in cold stresses responses [21]. This study proved that *GAUT* may be used as a candidate gene for future salt-resistant molecular breeding studies.

### 3.2. DEGs Involved in Calcium Signaling Pathway

Calcium (Ca^2+^), as the most critical second messenger, plays a vital role in the response of plants to abiotic stress. Stress signals immediately change the calcium level, and calcium signaling is then sensed and transduced through its downstream calcium-binding proteins. These processes can generate a series of biochemical reactions in plants to adapt to or resist various stresses. Ca^2+^ sensors mainly include calmodulin (CaM), calmodulin like protein (CML), calcium-dependent protein kinase (CDPK) and calcineurin B protein (CBL). CML plays a key role in stress signal transduction in plants. In this study, CML7 expression was up-regulated in the salt-tolerant cultivar T710MU under salt stress, but not in S710 (Table 1). It was reported that *AtCML9* can be induced by abiotic stress and ABA, and is involved in salt stress tolerance by regulating ABA-mediated pathways [22]. Similarly, the enhanced expression of CML*7* in the salt-tolerant cultivar T710MU illustrated that Ca^2+^ sensors participate in modulating salt stress tolerance in sugar beet. Furthermore, CBL protein interacting with calcium-independent protein kinase (CIPK) belongs to a Ca^2+^ mediated CBL-CIPK network in plant response to stress. In our study, *CBL2*, *CBL4* and *CBL7* were increased in the salt-tolerant cultivar T710MU under salt stress (Table 1). Salt overly sensitive (SOS) pathway is one of the most important signaling pathways in salinity signal transduction. AtCIPK24 can interact with AtCBL4 to regulate the Na^+^/H^+^ antiporter and increase salt tolerance in roots [23]. Moreover, AtCBL7 was found to be involved in regulating low-nitrate response in *Arabidopsis*, and the *cbl7* mutant exhibited lower nitrate content than wild-type plants under nitrate starvation condition [24]. Here, we report that these CBL genes are involved in the regulation of salt stress response in sugar beet.

### 3.3. DEGs Involved in Glycine, Serine and Threonine Metabolism

Amino acids play various roles in many life processes of plants. Besides their usage as protein constituents, they also influence a number of physiological processes such as signaling processes, synthesis of secondary metabolites and plant stress response [25]. In our study, a role for amino acids metabolism during salt stress in plants has been found, and several genes involving glycine, serine and threonine metabolism showed different expression patterns in the two sugar beet genotypes under salt stress (Table 1). Phosphoserine aminotransferase (SerC) is one of the key enzymes in biosynthesis of L-serine, and tryptophan synthase catalyzes the last step in the biosynthesis of tryptophan from indole and serine. In this study, *SerC* and *tryptophan synthase beta chain 1-like* were significantly induced in the salt-tolerant cultivar T710MU, but not in the salt-sensitive S710. The increasing expression of *SerB* and *tryptophan synthase beta chain 1-like* may enhance the synthesis of L-serine and tryptophan. It is reported that tryptophan was used as the precursor of melatonin synthesis [26]. Melatonin is thought to be an important phytohormone that enhances plant resistance to abiotic stress. 

In the glycine metabolism process, we found that choline monooxygenase and betaine-aldehyde dehydrogenase involved in glycinebetaine biosynthesis, were significantly up-regulated in the salt-tolerant cultivar T710MU (Table 1). A high accumulation of glycinebetaine in new leaves of sugar beet, especially under salt stress conditions, was previously observed [27]. Glycinebetaine clearly plays a key role in osmotic adjustment of sugar beet. Moreover, the levels of glycinebetaine in the salt-tolerant cultivar T710MU were higher than those in the salt-sensitive S710 (Figure 3). Therefore, these results demonstrated that the salt-tolerant cultivar enhances betaine synthesis through regulating gene expression-related glycine metabolism.

### 3.4. Other DEGs Involved in Salt Stress Tolerance 

Sugars not only act as the prime carbon and energy sources for most cell types, but also function in controlling metabolism, stress resistance, growth and development. Moreover, sugar metabolism in plants is closely associated with the response to salt stress [28]. Beta-glucosidases are a group of glycosyl hydrolases (GH) that are able to cleave the β-glucosidic linkages of di-or oligo-saccharides or other glucose conjugates, and they play a key role in activation of defense compounds [29]. In this study, *beta-glucosidases 18 (BGLU18*) was only identified to have increased expression in the T710MU plants (Table 2). Interestingly, *AtGLU18* were characterized as a glucosidase enzyme to hydrolyze glucose-conjugated ABA to release active ABA under plant abiotic stresses [30]. Therefore, the activation of inactive ABA pools by high level of AtGLU18 inT710MU is a strategy by which plants rapidly adjust ABA levels and respond to salinity environment.

Reactive oxygen species (ROS) produced under non-stress conditions play important roles as signaling molecules in plant growth and development. When plants were exposed to salt stress, the intracellular ROS homeostasis was destroyed, thereby increasing the production of ROS that could oxidize biological macromolecules such as lipids, proteins and carbohydrates. Plants also have the capacity to regulate ROS-generating and ROS-scavenging activities. For example, plants can use several antioxidant enzymatic sources to clear accumulated ROS. In our study, we found that cultivar T710MU maintains high antioxidant capacity to decrease ROS (Figure 3), which could induce oxidative damage in cells. POD plays an important role in plant growth, development and stress resistance. In this study, *POD7* and *SOD [Cu-Zn]* genes were significantly up-regulated in cultivar T710MU under salt stress, but not in cultivar S710 (Table 2). Overexpressing *POD* and *SOD [Cu-Zn]* in *Arabidopsis thaliana* was recently shown to enhance the defense response by regulating the accumulation of ROS and permeability of the leaf cuticle [31,32]. Moreover, the activities of POD and SOD in the salt-tolerant cultivar T710MU were higher than those in the salt-sensitive S710 under salt stress (Figure 2e). These genes may thus participate in regulating the tolerance of sugar beet to salt stress through reducing ROS level. 

Antimicrobial peptides (AMPs) are a group of molecules that are induced by plant fungi and bacteria, and they play important roles in innate immunity. Snakin is a short and cationic peptide with broad-spectrum antimicrobial activity that participates in the plant response to pathogen attack. *Snakin* expression has been reported to increase after fungal infection, wounding or methyl jasmonate exposure [33]. In addition, members of this family are involved in abiotic stress responses such as heat or oxidative stress. In our study, the *snakin* expression level was increased in the salt-tolerant cultivar under salt stress (Table 2). Therefore, snakin proteins may act as a regulator to modulate the response of plants to biotic or abiotic stress. Furthermore, another antimicrobial peptide, *vicilin-like 2–3*, was up-regulated in the salt-tolerant cultivar T710MU, whereas it maintained a stably expressed level in the salt-sensitive S710 under salt stress. A previous study indicated that some pathogen-related genes can provide enhanced tolerance against abiotic stress [34]. *Vicilin* may therefore be a promising candidate gene for developing multiple stress-tolerant sugar beet cultivars.

Auxin signaling plays a vital role in integrating a salt stimulus into plant development programs. Environmental stresses often affect auxin signaling by modulating Aux/IAA protein stability, and the sensitivity of root tissues to salt stress can be regulated by auxin transport [35]. In this study, several auxin signaling-related genes were differentially expressed under salt stress. Auxin transporter-like protein 4 was significantly induced in the salt-tolerant cultivar T710MU, but not in the salt-sensitive S710 (Table 2). Auxin transport and distribution regulated by the auxin transporter may affect the tolerance of sugar beet to salt. Furthermore, two *auxin-binding proteins 19a* (*ABP19a*) were up-regulated in cultivar T710MU but down-regulated or unchanged in S710 (Table 2). In *Arabidopsis*, ABP was found to bind auxin specifically and saturably, and it has been implied as a receptor for many auxin response ligands. *Arabidopsis* ABP1 protein also affects the expression of various cell wall remodeling genes [36]. In this study, increased *ABP19a* expression in cultivar T710MU may have been related to cell expansion and regulating the plant tolerance to salt stress.

## 4. Materials and Methods 

### 4.1. Plant Growth Conditions and Salt Stress Treatments

Seeds of sugar beet T710MU (salt-tolerant) and S710 (salt-sensitive) were stored in Heilongjiang University and identified by Gui Geng and Hui Liu. Two cultivars were grown in a greenhouse at the University. The culture conditions were as follows: a photoperiod of 14 h light/11 h dark for 24 h, day/night temperature of 25/20 °C, light intensity of 450 μmol m^−2^s^−1^ and relative humidity of 70%. Five days after germination, seedlings were transplanted to 1/2× Hoagland nutrient solution for 2 days. NaCl was gradually increased at a rate of 70 mM each day until the desired concentration was reached. Thereafter, a group of seedlings from both cultivars with uniform growth were treated with salt stress (1/2 × Hoagland solution with 280 mmol/L NaCl) for 15 days. Another group of seedlings was transferred to 1/2 × Hoagland nutrient solution (0 mmol/L NaCl) for 15 days. Each treatment group contained 12 seedlings (three biological replicates) for transcriptome sequencing and physiological indicator identification.

### 4.2. Determination of Physiological Indexes

The harvested seedlings were rinsed with ddH_2_O and dried with filter paper. Dry weight (DW) was determined after drying for 2 days at 80 °C. The chlorophyll content was measured as previously described [37]. The net photosynthetic rate was measured through an LC4 photosynthesis tester (ADC Bio Scientific). For MDA measurement, fresh leaves (0.5 g) were homogenized with 0.1% trichloroacetic acid (TCA) and centrifuged at 15,000 g for 10 min. An aliquot (1 mL) of the supernatant was mixed with 4 mL of 20% TCA prepared in 0.5% thiobarbituric acid (TBA) and incubated at 90 °C for 30 min. The reaction was then stopped and absorbance of the supernatant was measured at 532 and 600 nm. The method for determining the relative conductivity is the same as that reported in Wang et al. [38]. To determine antioxidant enzyme activity, 0.5 g of sugar beet leaves was ground in 3 mL of pre-cooled phosphate buffer, and the homogenate was centrifuged. The supernatant was designated as the crude antioxidant enzyme extract. SOD, CAT, POD, and APX activities were determined as previously described [2]. Betaine was extracted by grinding and homogenizing 0.5 g of the leaves in 3.5 mL of the buffer (methanol: trichloromethane: ddH_2_O, 12:5:3 *V/V/V*). The mixture was incubated at 65 °C and subsequently centrifuged for 15 min at 1200 g. The supernatants were adjusted to pH 6.0 and dried at 65 °C. The dried sample was dissolved in 1 mL of H_2_O, and the absorbance was recorded at 365 nm [39]. The proline content was determined according to the method of Ahmada et al. [40]. The free amino acid content was determined as previously described [39].

### 4.3. RNA Extraction, Library Preparation, and Sequencing 

Total RNA was extracted from leaves using TRIzol Reagent (Invitrogen, Carlsbad, CA, USA) according to the manufacturer’s instructions, and mRNA was enriched using the oligo (dT) magnetic bead method. cDNA libraries were constructed using the Truseq^TM^ RNA Sample Prep Kit (Illumina, San Diego, CA, USA) for the Illumina HiSeq PE150 high-throughput sequencing platform. The cDNA libraries were quantified using a TBS 380 fluorometer (Turner BioSystems, Sunnyvale, CA, USA). For each end of the produced paired-end sequences, 150 bases were sequenced.

### 4.4. RNA-Seq Data Analysis 

Raw data from the Illumina HiSeq platform were filtered to obtain high-quality clean reads, which included removing adaptor sequences, duplicated sequences, sequence quality values < Q20, reads containing more than 10% “N” (i.e., ambiguous bases in reads), reads < 70 bp in length after removing adapter sequences and reads where > 50% of the bases showed a Q-value ≤ 5. The clean sequences of each library were mapped to the reference sequence using TopHat (http://tophat.cbcb.umd.edu/). Thereafter, the expression levels of all genes were determined with the fragments per kilobase of exon model per million fragments mapped (FPKM) using Cufflinks software (http://cole-trapnell-lab.github.io/cufflinks/). Differentially expressed genes between samples were identified based on the FPKM value. FDR < =0.05 and log_2_ FPKM ratio > =1 and FPKM > =2 were set as thresholds to judge the significance of differences in gene expression. The Kyoto Encyclopedia of Genes and Genomes (KEGG) pathway database and the literature were used to determine the biological functions. For each sequencing library, SNP identification was conducted by identifying the mismatched bases between the reference genome and sequenced reads using amtools (http://samtools.sourceforge.net/) and VarScan v.2.2.7 (http://varscan.sourceforge.net/) software. The KEGG enrichment analysis of the differentially expressed genes (DEGs) was conducted using KOBAS software, and the KEGG pathways with *p*-values < 0.05 were assigned as significantly enriched pathways [41].

### 4.5. Quantitative Real-Time PCR (qRT-PCR) Analysis

Six genes were selected to validate the transcriptome analysis using qRT-PCR. Total RNA from the two cultivars was extracted with TRIzol reagent (Thermo Fisher Scientific, Waltham, MA, USA) according to the manufacturer’s instructions. For synthesis of the first strand of cDNA, a reverse transcription kit from TOYOBO (Japan) was used. Quantitative real-time PCR was performed using the Bio-Rad Quantitative PCR System (Bio-Rad, Hercules, CA, USA), and the *18s rRNA* gene served as internal gene. The PCR reaction system and procedure, as well as the concentration of SYBR GREEN, were consistent with our previous report [42]. All primer sequences are listed in Table S8 and each PCR reaction was performed twice with three replicates.

### 4.6. Statistical Analysis

All data were subjected to analysis of variance according to the model for completely randomized design using the SPSS 22.0 program (SPSS, Chicago, IL, USA). Differences among means ± SD (*n* = 3) of treatments were evaluated by the Duncan test at 0.05 probability level. 

## 5. Conclusions

**Figure 8:**
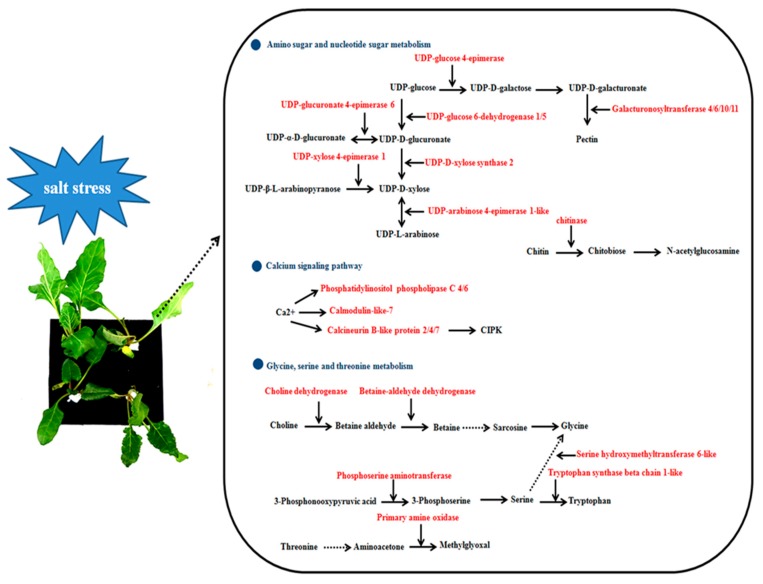
Schematic presentation of the potential mechanism of salt stress tolerance in T710MU tolerant sugar beet cultivar. The red highlighted genes indicate only up-regulation in salt-tolerant T710MU.

Our study investigates salt tolerance mechanisms in sugar beet based using a transcriptome approach with seedlings of contrasting genotypes. We have shown that sensitive and tolerant sugar beet cultivars respond differently to salt stress. The salt-tolerant nature of cultivar T710MU was probably due to the higher antioxidant enzyme activity and levels of osmotic adjustment molecules under stress conditions. Furthermore, DEGs in the two genotypes were compared to acquire important insights into the response mechanism specific to salt tolerance (Figure 8). Amino sugar and nucleotide sugar metabolism, calcium signaling pathway and glycine, serine and threonine metabolism are involved in sugar beet salt tolerance (Figure 8). The results indicate that transcriptome analysis of different genotypes under salt stress is an effective approach in improving our knowledge on salt tolerance in sugar beet.

## Figures and Tables

**Table 1:** List of selected genes for KEGG pathways in T710MU and S710.

	Gene ID	Annotation	T710MU Log_2_ (Fold Change)	S710 Log_2_ (Fold Change)
Amino sugar and nucleotide sugar metabolism	BVRB_9g210810	*UDP-D-xylose synthase 2*	+1.50283	No change
	BVRB_9g208380	*Galacturonosyltransferase10*	+1.38083	No change
	BVRB_5g120380	*Galacturonosyltransferase 6*	+1.91287	No change
	BVRB_8g184210	*Galacturonosyltransferase 4*	+1.31858	No change
	BVRB_5g113520	*Galacturonosyltransferase 11*	+1.15123	No change
	BVRB_9g212470	*Galacturonosyltransferase 3*	+1.01747	No change
	BVRB_5g113830	*UDP-glucose 6-dehydrogenase 1*	+2.96586	No change
	BVRB_9g204050	*UDP-glucose 6-dehydrogenase 1*	+1.69573	No change
	BVRB_7g169190	*UDP-glucose 6-dehydrogenase 5-like*	+1.69210	No change
	BVRB_3g051020	*Acidic endochitinase SE2*	+1.06524	No change
	BVRB_3g058210	*Acidic endochitinase SP2*	+4.00091	No change
	BVRB_5g109600	*UDP-arabinose 4-epimerase 1*	+1.34773	No change
	BVRB_5g100870	*UDP-xylose 4-epimerase 1*	+1.24388	No change
	BVRB_3g065540	*UDP-glucuronate 4-epimerase 6*	+1.06591	No change
	BVRB_8g189980	*Chitinase 6-like*	+3.43721	No change
Calcium signaling pathway	BVRB_9g207120	*Calmodulin-like-7*	+1.07209	No change
	BVRB_4g090720	*Calcineurin B-like protein 2*	+1.45304	No change
	BVRB_3g051930	*Calcineurin B-like protein 7*	+2.06100	No change
	BVRB_1g003040	*Calcineurin B-like protein 4*	+1.71420	No change
	BVRB_7g172070	*Phosphoinositide phospholipase C 4*	+2.26537	No change
	BVRB_2g023760	*Phosphoinositide phospholipase C 6*	+2.05940	No change
Glycine, serine and threonine metabolism	BVRB_2g026140	*Tryptophan synthase beta chain 1-like*	+2.3822	No change
	BVRB_6g130780	*Phosphoserine aminotransferase*	+1.36011	No change
	BVRB_6g143730	*Serine hydroxymethyltransferase 6-like*	+1.0856	No change
	BVRB_7g169880	*Betaine-aldehyde dehydrogenase 7*	+1.2431	No change
	BVRB_9g211640	*Choline monooxygenase*	+1.01747	No change
	BVRB_5g106360	*Glycine cleavage system H protein*	+1.62749	No change
	BVRB_6g148180	*Primary amine oxidase*	+1.23031	No change

“+” represents up regulation of gene expression.

**Table 2:** List of other genes involved in salt stress tolerance of sugar beet.

	Gene ID	Annotation	T710MU Log2 (Fold Change)	S710 Log2 (Fold Change)
	BVRB_4g087470	*Beta-glucosidase 18-like*	+2.87219	No change
	BVRB_9g214730	*Snakin-2-like*	+5.05443	No change
	BVRB_5g119620	*Vicilin-like antimicrobial peptides 2–3*	+3.99004	No change
	BVRB_2g029800	*Peroxidase P7-like*	+2.99716	No change
	BVRB_9g206190	*Superoxide dismutase [Cu-Zn]*	+1.15195	No change
	BVRB_3g058070	*Auxin-binding protein ABP19a-like*	+1.34462	No change
	BVRB_7g160890	*Auxin-binding protein ABP19a*	+13.1000	−1.49
	BVRB_5g111820	*Auxin transporter-like protein 4*	+4.38131	No change

“+” represents up regulation of gene expression; “−” represents down regulation of gene expression.

## Supplementary Materials

Supplementary Materials can be found at https://www.mdpi.com/1422-0067/20/23/5910/s1.
