# Supplementary material for: Transcriptome Analysis of Salt-Sensitive and Tolerant Genotypes Reveals Salt-Tolerance Metabolic Pathways in Sugar Beet
Source: Int J Mol Sci. 2019 Nov 25;20(23):5910. doi: 10.3390/ijms20235910 (PMC6928841; doi:10.3390/ijms20235910)
Supplement: Supplementary file 1 [file ijms-20-05910-s001.zip › Supplementary/Supplementary Table S1 and S8.docx]

**Table S1.** Sequencing statistics of 12 RNA libraries.

| **Sample** | **Number of raw reads** | **Number of Bases** | **Q20 (%)** | **Q30 (%)** | | **Number of high-quality clean reads** | **Reads mapped at high quality (%)** |
| --- | --- | --- | --- | --- | --- | --- | --- |
| S710- 0 mM NaCl-1 | 50884256 | 7632638400 | 96.70 | | 91.84 | 49281146 | 78.44% |
| S710- 0 mM NaCl-2 | 54471576 | 8170736400 | 96.36 | | 91.19 | 52436968 | 79.00% |
| S710- 0 mM NaCl-3 | 54808236 | 8221235400 | 96.56 | | 91.54 | 52973398 | 79.00% |
| S710- 280 mM NaCl-1 | 53325978 | 7998896700 | 96.72 | | 91.90 | 51672808 | 80.87% |
| S710- 280 mM NaCl-2 | 52139860 | 7820979000 | 96.51 | | 91.40 | 50388018 | 80.89% |
| S710- 280 mM NaCl-3 | 52080216 | 7812032400 | 96.71 | | 91.94 | 50408160 | 81.48% |
| T710MU- 0 mM NaCl-1 | 58405534 | 8760830100 | 96.42 | | 91.30 | 56298192 | 76.58% |
| T710MU- 0 mM NaCl-2 | 51669664 | 7750449600 | 96.27 | | 91.03 | 49703498 | 79.37% |
| T710MU- 0 mM NaCl-3 | 52489636 | 7873445400 | 96.51 | | 91.48 | 50683878 | 78.11% |
| T710MU- 280 mM NaCl-1 | 48318036 | 7247705400 | 95.65 | | 89.10 | 45765912 | 79.82% |
| T710MU- 280 mM NaCl-2 | 45801074 | 6870161100 | 96.52 | | 90.84 | 44057490 | 79.31% |
| T710MU- 280 mM NaCl-3 | 48298016 | 7244702400 | 96.21 | | 90.41 | 46361708 | 81.54% |

**Table S8.** List of the QRT-PCR primer

| **Primer name** | **Primer sequence** |
| --- | --- |
| *BVRB_9g210810-F* | 5-ATCGCATTCAGTTCCACCGT-3 |
| *BVRB_9g210810-R* | 5-ATGAAGGATCCTGGCGCAAA-3 |
| *BVRB_9g212470-F* | 5-TCCTAATGCTTGTGGCTGGG-3 |
| *BVRB_9g212470-R* | 5-CAACCGCGGCATTTCCTATG-3 |
| *BVRB_4g090720-F* | 5-CGCTCGTGGACTCTCTGTTT-3 |
| *BVRB_4g090720-R* | 5-ATGTGGCCACAACCATTTGC-3 |
| *BVRB_6g130780-F* | 5 AGGTGGGTTGGTTGAGGTTG-3 |
| *BVRB_6g130780-R* | 5-ACCATCTTCTCCTTGGCAGC-3 |
| *BVRB_9g211640-F* | 5-TCTGACACGAGGACCTTTGC-3 |
| *BVRB_9g211640-R* | 5- GAATGCAGGCCTCTGACAGT-3 |
| *BVRB_6g148180-F* | 5-GTGGAGCCAGAGAAGCATGT -3 |
| *BVRB_6g148180-R* | 5-CCTGTCGTGGAGGAAGCTTT -3 |
| *18S rRNA-F* | 5-AGCCCCTTCGAGCTCTTTTC-3 |
| *18S rRNA-R* | 5-CACTTCCTCGGCCACTTTCT-3 |
